# Therapeutic Role of Vitamin D in Multiple Sclerosis: An Essentially Contested Concept

**DOI:** 10.7759/cureus.26186

**Published:** 2022-06-22

**Authors:** Mahejabeen Fatima, Aselah Lamis, Shiza W Siddiqui, Tejaswini Ashok, Nassar Patni, Olatunji E Fadiora

**Affiliations:** 1 Internal Medicine, Deccan College of Medical Sciences, Hyderabad, IND; 2 Research, Dubai Medical College, Dubai, ARE; 3 Internal Medicine, JSS Medical College, Mysore, IND; 4 Internal Medicine, Deccan College Of Medical Sciences, Hyderabad, IND; 5 Internal Medicine, Windsor University School of Medicine, Cayon, KNA

**Keywords:** neuro-immunology, nutrition intervention, demyelinating dieases, neuro-degeneration, review of clinical trials, immune-mediated injury, serum vitamin d levels, multiple sclerosis

## Abstract

Multiple sclerosis (MS) is an immune-mediated demyelinating disease of the nervous system with incredibly intricate etiopathogenesis involving numerous genetic, epigenetic, and environmental risk factors. Major environmental risk factors include ultraviolet (UV) radiation, vitamin D, Epstein-Barr virus (EBV) infection, smoking, and high body mass index (BMI). Vitamin D, in particular, can be viewed as one piece of this puzzle, with various tabs and pockets, occupying a sequential site. In this article, we have briefly discussed the neuroimmunology of MS and the role of vitamin D in regulating immune responses. Various observational studies and clinical trials were reviewed and discussed according to stages of disease activity and course of the disease. The data reviewed in this article implied that serum vitamin D levels greatly influence the risk of developing MS and disease activity. Long-term follow-up studies indicated that low serum vitamin D levels correlate with worse disability outcomes. Since clinical trials did not provide significant evidence, the role of vitamin D in controlling disease activity remains unresolved. Larger clinical trials are needed to support the findings of observational studies and provide significant evidence in favour of vitamin D.

## Introduction and background

In May 1868, Jean-Martin Charcot, a French Neurologist, first assimilated complex clinical and pathological findings to establish a novel neurological disease - sclérose en plaques later referred to in English as Multiple Sclerosis (MS) [1]. Henceforth, it has been elaborated as an immune-mediated inflammatory demyelinating disease affecting the brain, spinal cord, and optic nerves. Worldwide, 2.8 million people live with MS, and the disease is four times more common in females than males [2]. It predominantly affects young adults and results in significant disability and decreased quality of life [2]. The most common form of the disease is the relapsing-remitting type (Relapsing-Remitting Multiple Sclerosis-RRMS); however, some people may have a progressive course from the onset (Primary Progressive Multiple Sclerosis-PPMS) or secondary to the initial relapsing course of the disease (Secondary Multiple Sclerosis-SPMS). 

Initial pathology involves inflammatory cell infiltration, demyelination, and axonal damage. Cumulative gliosis following repeated inflammation culminates in chronic neurodegeneration [3]. The first episode of inflammatory demyelination that may become clinical MS if further activity occurs later is identified as Clinically Isolated Syndrome (CIS) [4]. Jean-Martin Charcot's neurological triad, which includes intention tremor, nystagmus, and scanning speech, was one of the first clinical features of MS to be described. Other critical clinical features include visual dysfunction, diplopia, ataxia, sensory abnormality, spasticity, and bladder dysfunction [3]. This vast landscape of neurological manifestations results from variability in the site and extent of the lesion in the nervous system [1]. The aetiology of MS is a multifactorial process with genetic, lifestyle, and environmental factors playing a role in the causation of the disease [5]. Of the environmental factors, vitamin D, in particular, has piqued interest in recent years. The higher frequency of MS in places with less sunlight, low vitamin D levels linked to an increased risk of MS, and vitamin D's potential role in preventing autoimmune disorders all call for a closer study of its role in MS [6-8]. The purpose of this article is to review the protective abilities of vitamin D against autoimmunity and its implications in preventing and delaying the course of MS. 

## Review

Vitamin D and MS - immunopathological stance 

Immunologic Background of MS 

MS is an immune-mediated chronic inflammatory disease of the central nervous system (CNS). Attempts to understand the basis of inflammation over the years have presented us with two hypotheses. According to the “inside out” (CNS-intrinsic) hypotheses proposed by Stys et al. primary neuronal degeneration results in the loss of myelin and oligodendrocytes, as well as the release of CNS antigens into the periphery, resulting in an autoimmune inflammatory reaction [9]. The premise of the “outside-in” (CNS-extrinsic) hypothesis explains that the inflammatory trigger first activates immune cells at the periphery, after which then migrate to the CNS and cause demyelination. Both scenarios will result in a negative feedback loop: tissue damage causes antigens to be released to the periphery, which primes new immune responses in lymphoid tissue, followed by lymphocyte invasion into the CNS [10]. The pathogenesis involves dysregulated immune responses and includes cells of both innate and adaptive immune responses [11]. The role of Epstein-Barr virus (EBV) infection in the aetiology of MS is consistent with the CNS extrinsic hypothesis. Immunological evidence for this theory includes an increased risk of MS following EBV infection, molecular mimicry between EBV antigens and CNS antigens, and, high levels of anti-EBV antibodies in MS patients [12,13]. 

Vitamin D and Immune Function 

**Figure 1:**
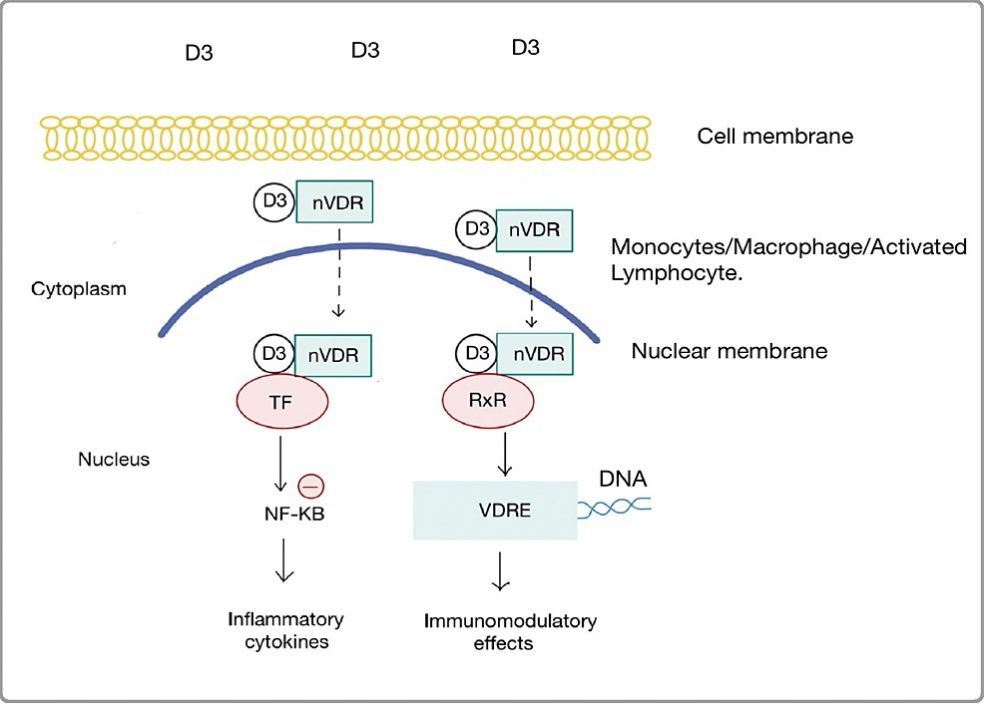
Figure showing intracellular mechanisms and immunomodulatory effects of vitamin D in immune cells. nVDR - Nuclear Vitamin D Receptor, TF - Transcription Factors, RxR - Retinoid X Receptor, NF-KB - Nuclear Factor Kappa B, VDRE - Vitamin D Responsive Elements, DNA - Deoxyribose Nucleic Acid Image credits- Mahejabeen Fatima

The primary site of production of the active form of vitamin D, calcitriol is in the proximal renal tubular cells. However, the *CYP27B1 *gene that codes for 1α hydroxylase enzyme which catalyzes the production of calcitriol (1,25(OH)2D) from 25(OH)D (major circulating form of vitamin D) is also expressed in cells outside of renal tissue where calcitriol is known to exert both autocrine and paracrine effects. Calcitriol and nuclear vitamin D Receptor (nVDR) complex bind to Vitamin D Responsive Elements (VRDEs) in the promoter sequences of DNA in monocytes, macrophages, and activated lymphocytes inducing differentiation, activation, and proliferation of immune and inflammatory cells [14]. This complex, after binding with transcription factors also regulates inflammatory mediators like tumor necrosis factor-α (TNF-α) and interferon-α (IFN-α) by inhibiting nuclear factor kappa B (NF-𝜅B) [15]. Calcitriol inhibits dendritic cell maturation by suppressing the expression of major histocompatibility complex (MHC) class II, cluster of differentiation (CD)40, CD80, and CD86. It decreases the production of pro-inflammatory cytokines like interleukin (IL)-12 and increases the synthesis of anti-inflammatory cytokines like IL-10 in dendritic cells. Calcitriol inhibits the production of IL-2, IL-17, and IFNs in T cells, as well as the cytotoxic activity and proliferation of CD4+ and CD8+ T cells. It promotes the production and activation of T regulatory cells and inhibits the production of B cell proliferation, differentiation, and immunoglobulin production [16]. The mechanisms through which vitamin D exerts these effects in immune cells are represented in Figure 1. 

Vitamin D as a therapeutic aid in MS

Role of Vitamin D in People at Risk of MS

Pathogenesis of MS involves a complex interplay amongst its genetic and environmental risk factors. However, environmental factors have the most significant impact and genetics only account for a small proportion of variations in the frequency of the disease. MS prevalence rises with increasing latitude among people of similar white ancestry all around the world [17]. Kurtzke, through his study of the epidemiology of MS, demonstrated that the risk for MS decreases two times with migration from high-risk to low-risk areas [18]. Latitude is known to be associated with numerous physical, chemical, biological, and social factors but, it is most tightly correlated with the duration and intensity of sunlight [19]. The findings of Kurtzke supplemented the results of early ecological studies which established an inverse relationship between sunlight exposure and the risk of MS. A multiple regression analysis done on a sample of 454 veterans with a diagnosis of MS born in 29 counties of the US in 1920 indicated that the risk of MS is inversely correlated with daily annual hours of sunshine (r = -0.73) and average December daily solar radiation at a place of birth (r = - 0.80) [20]. This effect of sunlight was thought to be largely due to the resultant increase in vitamin D levels in the body. But the direct relation of sunlight with vitamin D is eliminated in the context of race/ethnicity. Higher levels of serum 25(OH) are associated with decreased MS risk in whites but no such association was found in Blacks and Hispanics [21]. Increasing knowledge about the properties of ultraviolet (UV) radiation and its immunosuppressive effects has delineated sunlight among MS risk factors [22]. Lucas et al. performed a multicenter incident case-control study using a sample population of 611 people with a first demyelinating event (FDEs) between the age of 18 to 59 years residing in Australia (from latitudes 27°S to 43°S), from November 1, 2003, to December 31, 2006. The findings of this study revealed that higher levels of sun exposure (adjusted odds ratio {AOR} = 0.70, 95% confidence interval {CI}, 0.53-0.94) for each UV dose increment of 1,000 kJ/m^2^ (range 508-6,397 kJ/m^2^) and higher vitamin D levels (AOR = 0.93, 95% CI, 0.86-1.00) per 10 nmol/L increase in 25(OH)D) were independently associated with reduced risk of FDEs [23]. However, the impact of sunlight exposure through vitamin D cannot be entirely excluded. Vitamin D accounts for the 30% effect of sunlight on the risk of MS. Hedström et al. studied two population-based case-control studies and found that the effect of sunlight occurs through vitamin D and non-vitamin D pathways [24]. 

Sunlight is the most common source of vitamin D for most people. In areas of high MS prevalence, most of the sunlight is absorbed by the atmosphere and even sun exposure for a long time does not produce enough vitamin D. The relationship of latitude with the frequency of MS is inversed in areas predominated with cultures that consume vitamin D enriched diet. Kampman and Brustad observed that in Norway the average vitamin D consumption through food and supplements is equal to the current daily vitamin D intake recommendation for healthy people of 300 IU, which possibly explains why there is no evidence of latitude gradient in MS risk and implies a putative role of higher levels of vitamin D in reducing MS risk [25]. Outside of a normal diet, vitamin D intake through supplements is also associated with decreased MS risk. A large prospective cohort study performed on women taking multivitamins (vitamin D ≥ 400 IU/day) showed a 40% reduction in MS risk. However, the protective role of other vitamins included in multivitamins cannot be excluded [26]. Munger et al. performed a prospective nested case-control study on 257 US military personnel with the diagnosis of MS whose serum samples were stored in the Department of Defense Serum Repository. Estimation of vitamin D status of samples collected before the initial symptoms of MS showed a decreased risk of MS with increasing levels of 25(OH)D (OR for a 50-nmol/L increase in 25(OH)D, 0.59; 95% CI, 0.36-0.97). They also observed that this association was stronger for vitamin D levels measured before the age of 20 years [27]. The interactions of vitamin D with other environmental factors are more complex than previously known. A higher body mass index (BMI) is an established risk factor for MS. A recent Mendelian randomization (MR) analysis has provided evidence for this association. The relative contributions of BMI and low vitamin D to MS risk were explored. According to their findings, 5.2% of the increased risk of MS due to obesity is brought about by reduced vitamin D levels and vitamin D supplementation will marginally decrease the effect of obesity on MS [28]. 

VDREs are abundant in the promoter region of more than 80% of MS-susceptibility genes which when activated by vitamin D alters the expression of such genes [29]. Vitamin D interacts directly with HLA-DRB1*1501 which is the dominant haplotype of the *HLA-DRB1* locus found in Northern Europe [30]. It is possible that variations in vitamin D-related genes play a role in disease risk by altering serum levels of 25(OH)D. *CYP2R1 *gene codes for the 25-Hydroxylase enzyme that converts dietary vitamin D to 25(OH)D in the liver. A low-frequency genetic variation in this gene was identified through genome-wide association studies (GWAS) meta-analysis in the European population. This genetic variant is found to be associated with a two times increase in vitamin D insufficiency and a 40% increase in the risk of MS. Researchers tried to test this interaction between *CYP2R1 *and dietary vitamin D intake. They did not find any clear interaction in their assessment due to various limitations and proposed need for further study [31]. Similarly, various other vitamin D related genes involved in vitamin D synthesis, catabolism, and regulation of vitamin D metabolism can be responsible for increased MS risk by influencing serum vitamin D levels [30]. Various VDR single nuclear polymorphisms (SNPs) have been studied for their association with MS risk. These studies have shown some positive results but overall, the results are not univocal and remain inconclusive [32,33]. 

Observational studies have largely been the source of data establishing a link between vitamin D and MS risk. However, there are two notable drawbacks to these studies. The first is confounding, which may be eliminated by matching, and the second is reverse causation. These limitations are successfully overcome by MR analysis, which allows for the confirmation of a causal role of an exposure on an outcome using genetic variants as instrument variables. A large-scale MR analysis was performed using 25(OH)D-associated single nucleotide polymorphisms (SNPs) as instrument variables and 190 GWAS covering a broad range of diseases and human traits in order to establish a causal role of 25(OH)D. Their results indicate that a genetically predicted increase in 25(OH)D levels markedly reduced the risk of MS (OR = 0.824; 95% CI, 0.689-0.986) [34]. Another study performed by Wang yielded similar results. They used 20 25(OH)D instrument variables and the largest GWAS for 25(OH)D and MS to evaluate the effect of 25(OH)D levels on MS. They found an inverse link between serum 25(OH)D levels and MS risk using MR-egger (Beta = − 0.940, p = 0.001; OR = 0.391), weighted median (Beta = − 0.835, p = 0.000; OR = 0.434), inverse-variance weighted (IVW) (Beta = − 0.781, p = 0.000; OR = 0.458), simple mode (Beta = − 1.484, p = 0.016; OR = 0.227), and weighted mode (Beta = − 0.913, p = 0.000; OR = 0.401) (Table 1) [35]. The results of these recent studies have only reinforced the findings of earlier MR analyses [36-38]. 

**Table 1:** Summary of included studies showing the correlation between Vitamin D and MS risk FDE - First Demyelinating Event, CNS - Central Nervous System, GEMS - Genes and Environment in Multiple Sclerosis, EIMS - Epidemiological Investigation of Multiple Sclerosis, NHS - Nurses Health Study, RRMS - Relapsing-Remitting Multiple Sclerosis, VDR - Vitamin D Receptor, GWAS - Genome-Wide Association Studies, IEU - Integrative Epidemiology Unit, NHGRI-EBI - National Human Genome Research Institute-European Bioinformatics Institute Langer-Gould et al. [21]; Lucas et al. [23] ; Hedstrom et al., [24]; Munger et al., [26]; Munger et al., (2006) [27]; Harroud et al., [28]; Cancela Díez et al., [33]; Jiang et al., [34]; Wang R [35]

REFERENCES	DESIGN	SAMPLE SIZE	POPULATION	OBJECTIVES	CONCLUSIONS
Langer-Gould et al., (2018)	case-control	Blacks (116 cases/131 controls); Hispanics (183/197); whites (247/267)	members of Kaiser Permanente, Southern California	To examine the consistency of beneficial effects of 25OHD and/or sun exposure for MS risk across multiple racial/ethnic groups	Higher serum 25OHD levels were associated with a decreased risk of MS in whites but not in other racial groups.
Lucas et al., (2011)	case-control study	216 cases and 315 controls	people between the age of 18 to 59 years with an FDE living in Australia	measures of skin phenotype and actinic damage, and vitamin D status	Sun exposure and vitamin D status independently affect the risk of CNS demyelination.
Hedstrom et al., (2019)	case-control study	7069 cases and 6632 matched controls	GEMS study and EIMS study	To determine whether the influence of low sun exposure on MS risk is mediated by low vitamin D levels	Low sun exposure acts both directly on MS risk as well as indirectly, by leading to low vitamin D levels
Munger et al., (2004)	Prospective cohort study	NHS: 92,253 NHS II: 95,310	NHS and NHS II	To assess the protective effect of vitamin D on the risk of MS.	Intake of vitamin D supplements was associated with reduced MS risk. No such association was found with dietary vitamin D
Munger et al., (2006)	Prospective nested case-control study	Whites (148 cases, 296 controls) Blacks and Hispanics (109 cases, 218 controls)	More than 7 million US military personnel	To examine whether 25(OH)D levels are associated with the risk of MS	High circulating levels of vitamin D are associated with a lower risk of multiple sclerosis.
Harroud et al., (2021)	Mendelian randomization study	14,802 cases and 26,703 controls		The relative role of serum vitamin D levels and varying levels of adiponectin and leptin in the association between obesity and MS.	A minority of the increased risk of MS conferred by obesity is mediated by lowered vitamin D levels, while leptin and adiponectin had no effect
Cancela Díez et al., (2021)	Retrospective case-control study	209 cases and 836 controls	Patients with RRMS and healthy controls of Caucasian origin from Southern Spain.	To evaluate the association between polymorphisms in the VDR gene and the risk of MS	Only the VDR FokI (rs2228570) polymorphism was associated with developing MS.
Jiang et al., (2021)	Mendelian randomization study	Sample size ranged from 9,954 to 1,030,836 (median 112,561; mean 148,179)	190 GWAS from IEU OpenGWAS Project and NHGRI-EBI GWAS catalogue	To examine a causal role of vitamin D in various phenotypic traits and diseases	Genetically predicted 25(OH)D levels are inversely linked with the risk of MS
Wang R (2022)	Mendelian randomization study	14,498 cases and 24,091 controls	GWAS of European ancestry	To examine a causal role of vitamin D in MS risk	A causal link between genetically predicted serum 25(OH)D levels and MS risk

Role of Vitamin D in RRMS

RRMS is the most common form of the disease. It involves a more active course of inflammation with disruption of the blood-brain barrier by activated microglia and infiltration of immune cells. The activity of these immune perpetrators in the neuronal tissue results in demyelinated plaques. This type of acute inflammation has a tendency to recur in the course of the disease [39]. A population-based prospective cohort study performed on 145 people with RRMS residing in Southern Tasmania and Australia from 2002 to 2005 showed that higher 25(OH)D levels were associated with decreased risk of relapse. Each 10 nmol/L increase in 25(OH)D resulted in up to a 12% decrease in relapse risk [40]. These clinical findings also correlate with radiological signs of MS disease activity. A prospective cohort study conducted among 1482 participants with MS in the BEYOND study treated with interferon beta-1b showed that an increase in blood 25(OH)D levels of 50.0 nmol/L were linked to a 31% lower rate of new lesions on MRI (relative rate {RR}, 0.69; 95% CI, 0.55-0.86; P =.001). Patients with 25(OH)D levels more than 100.0 nmol/L had the lowest rate of new lesions on MRI (RR, 0.53; 95% CI, 0.37-0.78; P =.002), however, no significant association with relapse rates was observed [41]. In a large multicenter study that followed 1047 clinically isolated syndrome (CIS) cases from 17 different countries for a median of 4.31 years, clinical and biochemical variables were examined to establish their value in predicting CIS advancing to clinically definite multiple sclerosis (CDMS). In univariable analysis, it was observed that the risk of conversion was significantly reduced in patients with high 25(OH)D levels. The same results were not replicated in multivariable analysis where the risk of conversion was decreased but the statistical significance was also diminished simultaneously [42]. 

Although there is mounting evidence in favour of vitamin D reducing MS risk obtained through observational studies, the same has not been proved conclusive in clinical trials. Mc Laughlin et al. performed a meta-analysis on 12 clinical trials assessing vitamin D in patients with RRMS. Randomized control trials (RCTs) that reported relapse rates, change in Expanded Disability Status Scale (EDSS) and appearance of new radiological signs were selected exclusively. The results of this meta-analysis showed that vitamin D supplementation might be beneficial in preventing relapse rates and new radiological signs. For changes in EDSS, this relationship was found to be relatively strong. However, these changes were not statistically significant for any outcome measure (Table 2) [43]. If the reported correlations in the observational studies could be directly scaled up, this would imply that vitamin D has a massive treatment effect, almost completely eliminating disease activity. The interpretation of data from randomised controlled trials (RCTs) has not proved the same [44]. Possible explanations for this disparity are confounding and reverse causality in observational studies that may have overestimated the influence of vitamin D. RCTs are powered by observational studies and therefore the power calculated for RCTs on vitamin D may not be sufficient enough to detect less prominent effects of vitamin D [44]. 

**Table 2:** Summary of studies included assessing the relation between serum vitamin D levels and disease activity RRMS - Relapsing-Remitting Multiple Sclerosis, CIS - Clinically Isolated Syndrome, CDMS - Clinically Definite Multiple Sclerosis Simpson Jr et al., [40]; Fitzgerald et al., [41]; Kuhle J et al., [42]; Mc Laughlin et al., [43]

REFERENCES	DESIGN	SAMPLE SIZE	POPULATION	OBJECTIVES	CONCLUSION
Simpson Jr et al., (2010)	Prospective cohort study	145	Residents of Southern Tasmania and Australia from 2002 to 2005 with RRMS	To observe relapse rates in patients of RRMS with higher levels of 25(OH)D	12% decrease in relapse risk for each 10nmol/l increase in serum 25(OH)D was observed
Fitzgerald et al., (2015)	Prospective cohort study	1796	Patients with RRMS and on IFN- β therapy from 26 countries	To assess the association between 25(OH)D and disease activity and progression	A 50.0-nmol/L increase in serum 25(OH)D levels was associated with a 31% lower rate of new lesions
Kuhle et al., (2015)	Multicenter study	1047	Patients with CIS from 17 different countries	Predictive value of 25(OH)D in CIS to CDMS conversion	Significant predictive value in univariate analysis. Diminished significance in multivariable analysis
Mc Laughlin et al., (2018)	Meta-Analysis	950	Participants of 12 clinical trials	To determine the therapeutic role of vitamin D in MS	Statistically insignificant differences in outcome measures

Role of Vitamin D in Progressive MS

Patients with progressive MS develop diffuse inflammation involving white matter and the cortex along with chronically demyelinated axons. Effective repair of demyelinated axons following acute inflammation will reduce the burden of disability in these patients [45]. The University of Cambridge reported that vitamin D activates the nVDR, which when paired with the retinoid X receptor (RXR-) leads to downstream signalling and increased differentiation of oligodendrocyte (OLG) progenitor cells into mature OLGs, which are important for myelin synthesis around neurons and are commonly damaged cells in MS. The study also discovered vitamin D receptor expression in oligodendrocyte lineage cells in MS and its activation leading to increased OLG differentiation [46,47]. An experimental study performed on rats has provided support for this hypothesis. Twenty-four male Wistar rats with surgical lesions to the corpus callosum were divided into three groups. Rats from two out of three groups were given lysolecithin to induce toxic demyelination and rats from one out of three groups were supplemented with vitamin D following lysolecithin administration. It was observed that vitamin D administration resulted in a proliferation of neuronal stem cells which later differentiated into OLG lineage cells [48]. 

Ascherio et al. followed up participants of the BENEFIT study with CIS for 5 years clinically and by MRI. According to their observations higher 25(OH)D levels were associated with a slower rate of progression, 25% lower yearly increase in T2 lesion volume (P < .001), and 0.41% lower yearly loss in brain volume (P = .07) from months 12 to 60. 25(OH)D levels greater than or equal to 50 nmol/L (20 ng/mL) predicted lower disability (Expanded Disability Status Scale score, -0.17; P =.004) over the next four years. Lower vitamin D levels in the initial disease course in patients with MS being treated with interferon beta 1-b are associated with long-term MS activity and disease progression (Table 3) [49]. An 11-year follow-up study of the BENEFIT trial was performed by Cortese et al. to examine the effects of vitamin D, smoking, and EBV antibody titers on the cognitive status of patients with MS. According to their results patients with lower vitamin D levels during the course of disease activity are inclined towards worse long-term cognitive function [50]. A similar long-term study was performed by Wesnes et al. where they explored the influence of vitamin D levels, tobacco use, and BMI on disability progression in patients with MS. These parameters were repeatedly measured in 88 patients with RRMS who completed a randomized controlled study on ω-3 fatty acids between 2004 and 2008. EDSS is a tool used to assess clinical disability in MS patients. At follow-up after 10 years in 2017, clinical disability was measured using EDSS among 80 participants. Higher serum 25(OH)D levels were associated with lower 10-year EDSS progression after adjusting for potential confounders [51]. Moreover, self-reported vitamin D supplement use was linked to higher physical and mental quality of life (QoL) cross-sectionally, but only with increased physical QoL prospectively. A study in which data from the HOLISM international cohort was reviewed at 2.5 years indicated higher QoL scores in patients taking vitamin D at an average daily dose of over 50,000IU/d [52]. 

**Table 3:** Summary of included studies assessing the effect of vitamin D on long term outcomes of MS CIS - Clinically Isolated Syndrome, BENEFIT - Betaferon / Betaseron in Newly Emerging Multiple Sclerosis for Initial Treatment, EBV - Ebstein Barr Virus, RRMS - Relapsing-Remitting Multiple Sclerosis, BMI - Body Mass Index, HOLISM - Health Outcomes and Lifestyle In a Sample of People with Multiple Sclerosis, QoL - Quality of Life Ascherio et al., [49]; Cortese et al., [50]; Wesnes et al., [51]; Simpson-Yap et al., [52]

REFERENCES	SAMPLE SIZE	POPULATION	OBJECTIVE	CONCLUSION
Ascherio et al., (2004)	468	Patients with CIS from 18 European countries, Israel and Canada, participants of the BENEFIT trial	To evaluate serum25 (OH)D levels as a prognostic marker for MS outcomes in patients with CIS	Higher serum 25(OH)D levels were associated with reduced disease activity and rates of progression
Cortese et al., (2020)	278	Patients with CIS from 18 European countries, Israel and Canada, participants of the BENEFIT trial	To assess the effect of serum vitamin D levels, smoking and anti EBV antibody concentrations on long term cognitive status in MS patients	Reduced vitamin D levels and smoking during the course of disease results in worse outcomes
Wesnes et al., (2021)	88	Patients with RRMS aged between 18 and 55 years in Norway	To determine the association of vitamin D, tobacco use and BMI with disability progression in MS	Only lower vitamin D levels were associated with worse long term disability progression in MS
Simpson-Yap et al., (2021)		Participants of the HOLISM international cohort	To examine the effect of sun exposure and vitamin D on quality of life	Vitamin D supplement use was linked to higher physical and mental quality of life cross-sectionally, but only with increased physical QoL prospectively.

Prognostic value of vitamin D in MS

It may be implied that vitamin D while being involved in causation also influences the course of the disease and long-term disability outcomes [26,34]. While causality was established through Mendelian randomization studies, observational studies showed that lower levels of 25(OH)D were linked to increased risk of disease, higher rates of relapses, and worse long-term outcomes. However, clinical trials did not prove this influence to be true. Clinical trials performed thus far may have shown some benefit of 25(OH)D on disease activity but they did not prove any significant effect on the primary outcome of the trials. Particularly, the studies performed to assess the effect of vitamin D on disease activity did not provide such evidence. 

Limitations

Multiple sclerosis is a multifactorial disease and this article focuses only on one entity of its etiopathogenesis. As mentioned in this article, vitamin D interacts with other environmental and genetic factors of the disease. Therefore, its effect on the disease risk, activity, and progression cannot be entirely delineated. A small proportion of studies showing contradictory results pertaining to the current topic have not been included in this review. 

## Conclusions

Vitamin D appears to have numerous associations with various etiological factors of MS. Most of the MS susceptibility genes have been known to closely interact with vitamin D. It is directly associated with disease risk and activity due to its homeostatic role in the immune system. Mendelian randomization studies have also proved a causal relationship between low vitamin D levels and the risk of MS. Mutations in genes that code for the enzymes involved in the metabolic pathway of vitamin D synthesis as well as degradation and VDR SNPs are also implicated in the aetiology of MS. Various epidemiological and observational studies have proposed that higher levels of vitamin D are linked with decreased risk of relapses and slower progression of the disease. Clinical trials due to some limitations have largely been inconclusive about the effect of vitamin D on disease activity. Therefore, the use of vitamin D, as opposed to current highly efficacious disease-modifying treatments, remains unproven. Larger placebo-controlled randomized control trials are needed in order to endorse vitamin D supplementation as focal therapy in the treatment of MS.
